# The impact of the COVID-19 pandemic on referrals to musculoskeletal services from primary care and subsequent incidence of inflammatory rheumatic musculoskeletal disease: an observational study

**DOI:** 10.1093/rap/rkad044

**Published:** 2023-05-02

**Authors:** Claire Burton, Ram Bajpai, Kayleigh J Mason, James Bailey, Kelvin P Jordan, Christian D Mallen, Victoria K Welsh

**Affiliations:** Centre for Musculoskeletal Health Research, School of Medicine, Keele University, Keele, UK; Centre for Musculoskeletal Health Research, School of Medicine, Keele University, Keele, UK; Centre for Musculoskeletal Health Research, School of Medicine, Keele University, Keele, UK; Centre for Musculoskeletal Health Research, School of Medicine, Keele University, Keele, UK; Centre for Musculoskeletal Health Research, School of Medicine, Keele University, Keele, UK; Centre for Musculoskeletal Health Research, School of Medicine, Keele University, Keele, UK; Centre for Musculoskeletal Health Research, School of Medicine, Keele University, Keele, UK

**Keywords:** COVID-19, inflammatory rheumatic and musculoskeletal disease/disorders, electronic health-care record research

## Abstract

**Objective:**

The aim was to describe the impact of the COVID-19 pandemic upon referral patterns and incident diagnosis of inflammatory rheumatic and musculoskeletal diseases (iRMDs).

**Methods:**

UK primary care data were used to describe referral patterns for patients with musculoskeletal conditions. Trends in referrals to musculoskeletal services and incident diagnoses of iRMDs (specifically, RA and JIA) were described using Joinpoint Regression and comparisons made between key pandemic time periods.

**Results:**

The incidence of RA and JIA reduced by −13.3 and −17.4% per month, respectively, between January 2020 and April 2020, then increased by 1.9 and 3.7% per month, respectively, between April 2020 and October 2021. The incidence of all diagnosed iRMDs was stable until October 2021. Referrals decreased between February 2020 and May 2020 by −16.8% per month from 4.8 to 2.4% in patients presenting with a musculoskeletal condition. After May 2020, referrals increased significantly (16.8% per month) to 4.5% in July 2020. The time from first musculoskeletal consultation to RA diagnosis and from referral to RA diagnosis increased in the early pandemic period [rate ratio (RR) 1.11, 95% CI 1.07, 1.15 and RR 1.23, 95% CI 1.17, 1.30, respectively] and remained consistently higher in the late pandemic period (RR 1.13, 95% CI 1.11, 1.16 and RR 1.27, 95% CI 1.23, 1.32, respectively), compared with the pre-COVID-19 pandemic period.

**Conclusion:**

Patients with underlying RA and JIA that developed during the pandemic might be yet to present or might be in the referral and/or diagnostic process. Clinicians should remain alert to this possibility, and commissioners should be aware of these findings, enabling the appropriate planning and commissioning of services.

Key messages

- Primary care has continued to refer patients with suspected iRMDs after an average of three musculoskeletal-related consultations, throughout the pandemic.
- The reduced incidence of RA seen during the pandemic period suggests that there remains a group of patients with undiagnosed RA.
- Residents of deprived communities waited longer for their diagnosis once referral had been made.

## Introduction

Rheumatic and musculoskeletal diseases (RMDs), defined as problems of the joints, muscles and bones [1], are common and cause a high disease burden globally [2]. In the UK, primary care is generally the first point of care for people with RMDs; 20% of adults consult their primary care clinician with an RMD each year [3]. Patients are typically managed using a multidisciplinary approach that includes advice and self-care, pharmacological therapy and referral for non-pharmacological treatments, such as physiotherapy, exercise and weight management. However, inflammatory rheumatic musculoskeletal diseases (iRMDs), including RA, JIA and other autoimmune and inflammatory conditions (including PsA, axial spondyloarthropathies, SLE, PMR and GCA), require rapid diagnosis and management, often involving specialist care referral.

In March 2020, the coronavirus disease 2019 (COVID-19) pandemic prompted the delivery of UK primary care to change abruptly [4]. The ‘total triage’ model of care, whereby all consultations were ‘remote by default’, was widely implemented [5, 6], replacing traditional ‘face-to-face’ health care. The Government issued a ‘stay at home’ order, advising the public to ‘protect the NHS’, and instigated a ‘lockdown’. [7] Except for emergency or urgent cases, including suspected cancer referrals, primary care clinicians were encouraged to reduce referrals to community and secondary care to enable increased acute care capacity [8, 9]. These restrictions led to a ∼30% reduction in primary care consultations per person in the week after introduction of the lockdown, with generally lower consultation rates observed until June 2020 [4]. Delays in the diagnosis and treatment of cancers ensued [10], and similar concerns were expressed regarding other time-critical diagnoses.

Early diagnosis of iRMDs and subsequent timely access to disease-modifying therapies are associated with improved health and socio-economic outcomes [11] and form a key component of national and international guidelines [12, 13]; for example, timely referral within 3 working days of presentation with suspected RA symptoms to primary care [12]. The UK mandatory National Early Inflammatory Arthritis audit includes standards for referral to rheumatology (3 days), being seen by a rheumatologist (3 weeks) and being initiated on treatment (<6 weeks of referral) [14]; a 14-month audit suspension started in March 2020.

We used routinely collected, anonymized, primary care data to describe the impact of the COVID-19 pandemic upon time to referral and diagnosis iRMDs, including RA and JIA, in patients presenting to primary care with musculoskeletal symptoms.

## Methods

### Setting, study population and time line

Longitudinal routinely collected electronic primary care record data from the Clinical Practice Research Datalink (CPRD) Aurum database were analysed from 1 April 17 to 31 October 2021. CPRD Aurum contains information from >13 million current patients (20% of the UK population), including children and adults, from 15% of all UK general practices, and is broadly representative of primary care in England and, since 2019, Northern Ireland [15]. No exclusion criteria were applied; all included patients had at least one musculoskeletal consultation during the observed period. The study considered three time periods: pre-COVID-19 pandemic, 1 April 2017–31 March 2020; early COVID-19 pandemic, 1 April 2020–31 July 2020; and late COVID-19 pandemic, 1 August 2020–31 October 2021. The study was approved by the CPRD Independent Scientific Advisory Committee (protocol number 20_141) and access granted to the data required for the study. Linked data were obtained for the patient-level index of multiple deprivation (IMD).

### Case definition, preceding musculoskeletal consultations and referral codes

Comprehensive code lists based on previous published work [3] and updated with SNOMED CT codes [16] that were introduced to primary care from April 2018, were developed (DOI 10.17605/OSF.IO/RJ56X), through a consensus exercise between practising clinicians.

Cases of iRMDs were inclusive of diagnoses including RA, PsA, inflammatory spondyloarthritidies, CTD (e.g. SLE, scleroderma), PMR and gout. The iRMD codes were grouped together and subcategorized into RA and JIA. Consultations relating to a musculoskeletal (synonymous with RMD) condition, defined as a symptom, condition or disease relating to the joints, muscles and bones, had been identified previously using a similar broader code list, with exclusion of trauma-related entries. Codes relating to referrals were inclusive of rheumatology, musculoskeletal interface service, orthopaedics and pain services.

### Analysis

The incidence of iRMDs was defined as the total number of patients with an identified code for an iRMD who had no previously recorded code in their record, divided by the total registered population without an iRMD diagnosis at the start date.

The proportion of patients who were referred to specialist musculoskeletal services for the first time was calculated per 100 musculoskeletal consulters, in each calendar month. Crude incidences of all iRMDs, RA and JIA were calculated as per 100 000 registered population (per 1 000 000 for JIA, owing to the small numbers). Temporal trends were determined by calculating the mean monthly percentage change (MPC) using Joinpoint Regression analysis in Joinpoint Program v.4.9.10. A positive value of MPC suggests an increasing trend, whereas a negative value suggests a decreasing trend. Optimal models (<5 Joinpoints) were selected using a permutation test with 4500 permutations, with a significance level of *P* < 0.05.

For all iRMDs, RA and JIA categories, four outcomes were defined: time from first (non-traumatic) musculoskeletal consultation to ‘incident iRMD’ diagnosis; first musculoskeletal consultation (in the preceding 24-month period, of any mode) to first musculoskeletal referral; first musculoskeletal referral to ‘incident iRMD’ diagnosis; and number of consultations between first musculoskeletal consultation and referral/‘incident iRMD’ diagnosis (noting that not all diagnoses were preceded by a referral). These outcomes were reported as median values with the interquartile range (IQR) and stratified by geographical region (England: East Midlands; East of England; London; North East; North West; South Central; South East Coast; South West; West Midlands; Yorkshire and the Humber; and Northern Ireland) and IMD quintiles (Q1: least deprived; Q5: most deprived). Age- and sex-adjusted incidence rate ratios (RRs) were calculated using negative binomial regression and reported with 95% CIs for each outcome. To ensure that all patients with a coded iRMD diagnosis were considered in the analysis, individuals were included in the time period within which their outcome occurred. For example, if they presented to primary care in the pre-pandemic period and were diagnosed with an iRMD in the late pandemic period, they would be included as a patient in the late pandemic period.

### Patient and public involvement

Patient and public involvement began after the research question was developed. Patient and public involvement added context to statistical results by reflecting upon personal experiences of accessing primary care for RMD symptoms during the pandemic and navigating the wider NHS environment. Patient and public involvement helped to identify study limitations and develop the co-production of further research questions.

## Results

The numerator population (individuals presenting with an iRMD-related primary care consultation) included 6 057 747 patients from practices in England and Northern Ireland. The denominator population remained largely constant over the course of the study period at ∼13.5 million individuals.

**Figure 1.**
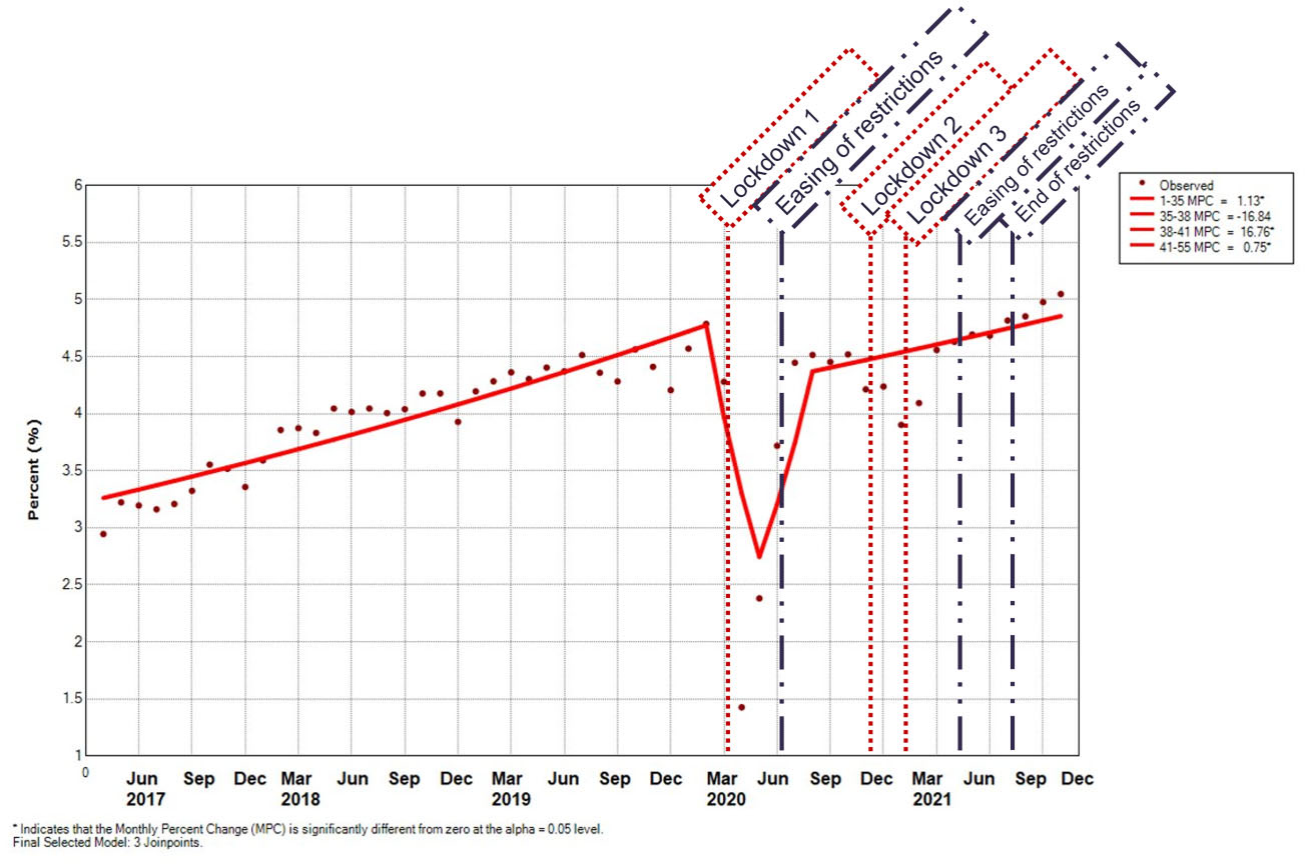
Proportion of patients referred to specialist musculoskeletal services for the first time, per 100 musculoskeletal consulters. Predicted values were modeled using joinpoint regression analysis

The prevalence and incidence of musculoskeletal consultations (and associated prescribing) have been described, and demonstrated reduced rates in the early COVID-19 pandemic period, with recovery through the late COVID-19 pandemic period (DOI 10.37361/n.2021.1). Fig. 1 (and associated Supplementary Table S1, available at *Rheumatology Advances in Practice* online) demonstrates that the proportion of patients with an eventual diagnosis of an iRMD, presenting to primary care with any non-traumatic RMD, who had an incident referral to musculoskeletal services in each given month, had been increasing from 2.9 to 4.8% [MPC 1.1 (95% CI 1.0, 1.3)] between April 2017 and February 2020. This rate then decreased between February 2020 and May 2020 to 2.4% [MPC −16.8 (95% CI −35.5, 7.2)]. Around the first easing of UK lockdown restrictions, the proportion of patients referred began to increase again to pre-pandemic levels and to 4.5% in August 2020 [MPC 16.8 (95% CI 0.2, 36.1)], then at a slower rate of 0.8 MPC (95% CI 0.1, 1.4) until October 2021, with 5.1% of patients having a consultation for a musculoskeletal problem referred that month. There was a discernable dip in data points in January 2021 to 3.9% at the time of the third lockdown; however, this did not force a Joinpoint in the model.

**Figure 2.**
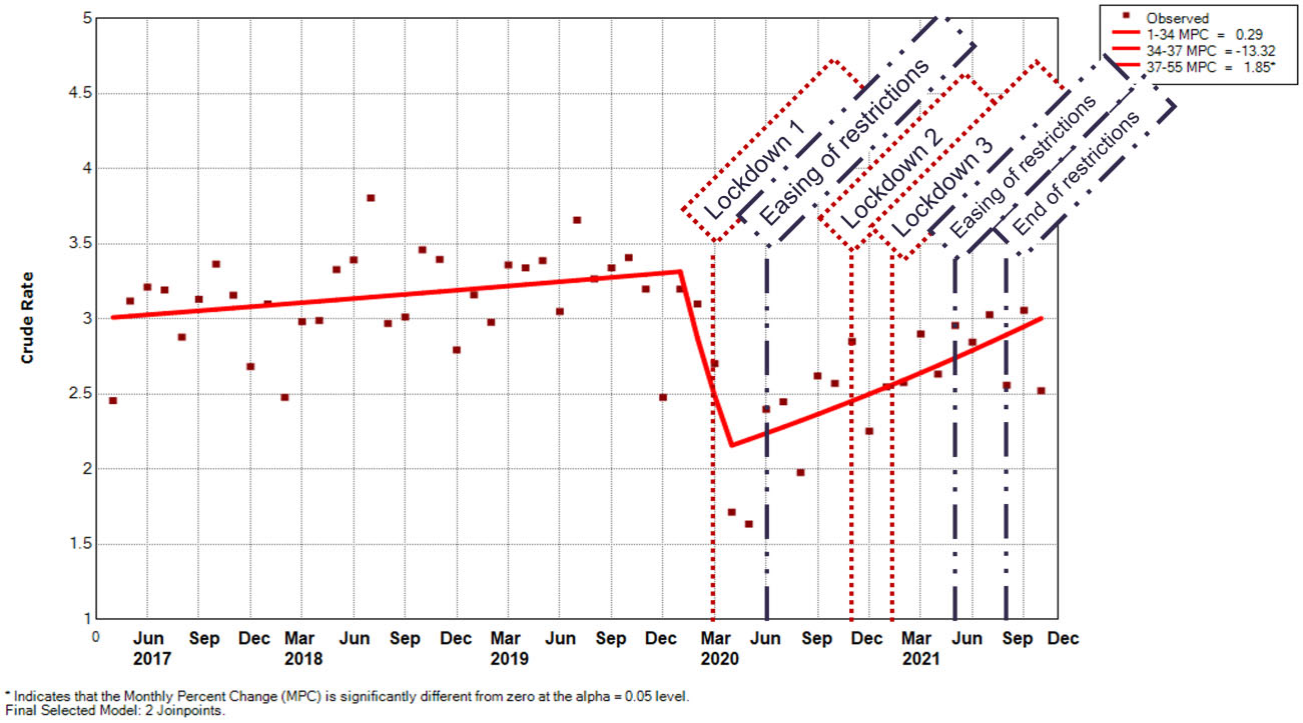
Incidence rate per month of RA per 100 000 registered population. Predicted values were modeled using joinpoint regression analysis

In line with referrals, RA incidence had been increasing from 2.5 (95% CI 2.2, 2.7) to 3.2 (2.9 to 3.5) per 10 000 registered population [MPC 0.3 (95% CI −0.1, 0.7)] from the start of the observed period until January 2020 (Fig. 2; Supplementary Table S2, available at *Rheumatology Advances in Practice* online). The rate then decreased during the first lockdown to 1.7/10 000 (95% CI 1.5, 1.9) in April 2020 [MPC −13.3 (95% CI −35.4, 16.3)], before starting to increase again back to 2.5/10 000 (95% CI 2.3, 2.8) [MPC 1.9 (95% CI 0.8, 2.9)], until the end of the observation period in October 2021.

**Figure 3.**
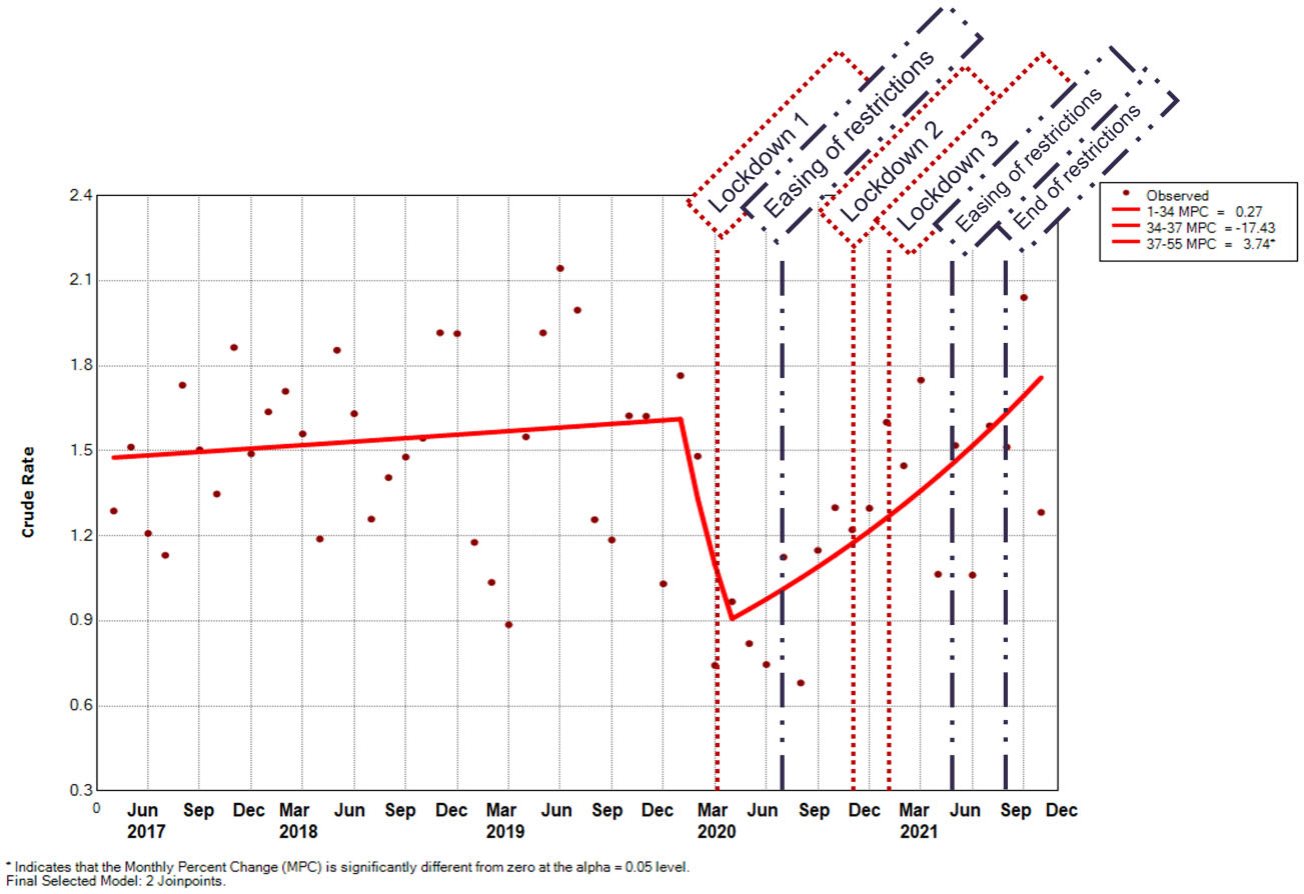
Incidence rate per month of JIA per 1 000 000 registered population. Predicted values were modeled using joinpoint regression analysis

Fig. 3 and Supplementary Table S3, available at *Rheumatology Advances in Practice* online, demonstrate that the incidence of JIA was stable between the start of the observation period and January 2020, at 1.3 per 1 000 000 registered population (95% CI 0.7, 1.9) to 1.8 (95% CI 1.1, 2.5) [MPC 0.3 (95% CI −0.5, 1.0)], followed by a decrease until March 2020, when the rate was 0.7 (95% CI 0.3, 1.2) [MPC −17.4 (95% CI −59.4, 68.1)]. Of note, March 2020 is where the Joinpoint lies; however, the data points show a reduction into August 2020, when the incidence was 0.7 (95% CI 0.2, 1.1). There was then an increase in incidence until the end of the observed period in October 2021 to 1.3 (95% CI 0.7, 1.9) [MPC 3.7 (95% CI 1.6, 6.0)].

**Figure 4.**
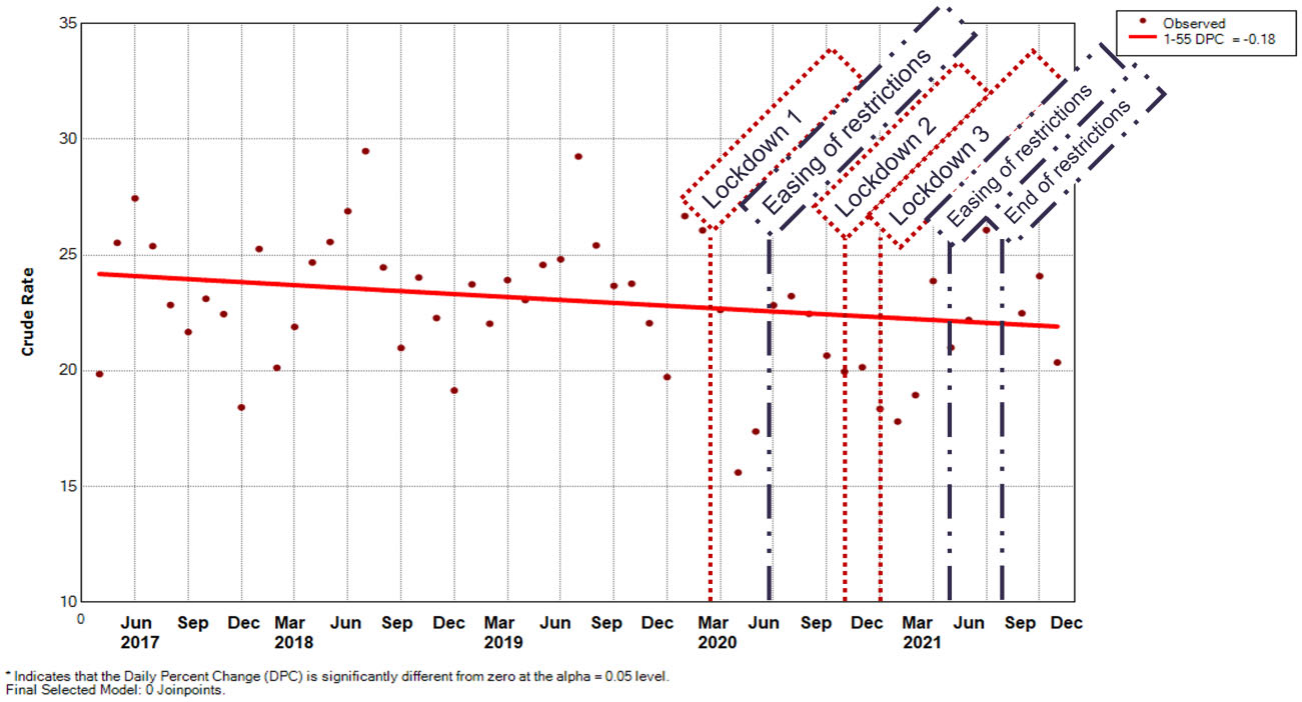
Incidence rate per month of all inflammatory rheumatic musculoskeletal disease per 100 000 registered population. Predicted values were modeled using joinpoint regression analysis

The incidence of all iRMDs did not change significantly over time. Fig. 4 and Supplementary Table S4, available at *Rheumatology Advances in Practice* online, demonstrate a gradual increase in the incidence from 19.9 (95% CI 19.1, 20.6) to 20.4 (95% CI 19.6, 21.1) [MPC −0.2 (95% CI −0.4, 0.0]. Supplementary Table S5, also available at *Rheumatology Advances in Practice* online, details crude monthly estimates for referral and incidence rates.

Table 1 demonstrates that not all patients with a coded incident diagnosis of RA/JIA/iRMD had a coded referral. Around 20% of patients who received a diagnosis of RA did not have a referral code, ∼50% of patients with a diagnosis of JIA did not have a referral code, and ∼60% of patients with a diagnosis of any iRMD did not have a referral code. These percentages were not substantially different between pandemic periods.

**Table 1.** Comparison of time between key events pre-COVID-19 and during early and late COVID-19 pandemic periods

Outcomes by condition set	Pre-COVID-19 pandemic period (1 April 2017–31 March 2020)		Early COVID-19 pandemic period (1 April 2020–31 July 2020)		Late COVID-19 pandemic period (1 August 2020–31 October 2021)		Pre- *vs* early COVID-19 pandemic period	Pre- *vs* late COVID-19 pandemic period
	Number of patients	Median number of days (IQR)	Number of patients	Median number of days (IQR)	Number of patients	Median number of days (IQR)	RR (95% CI)	RR (95% CI)
RA								
First musculoskeletal consultation to RA diagnosis, days	32 627	1026 (405, 1443)	2510	1237 (470, 1596)	5865	1264 (515, 1649)	1.11 (1.07, 1.15)	1.13 (1.11, 1.16)
First musculoskeletal consultation to first referral, days	24 842	448 (77, 1026)	2034	458 (53, 1099)	4665	438 (59, 1091)	1.02 (0.96, 1.09)	1.03 (0.99, 1.08)
First referral to RA diagnosis, days	24 842	196 (63, 673)	2034	274 (64, 910)	4665	287 (69, 932)	1.23 (1.17, 1.30)	1.27 (1.23, 1.32)
Number of consultations between first musculoskeletal consultation and referral/RA diagnosis	30 143	3 (1, 6)	2338	3 (1, 5)	5486	3 (1, 5)	0.92 (0.88, 0.96)	0.92 (0.90, 0.95)
JIA								
First musculoskeletal consultation to JIA diagnosis, days	1277	409 (102, 1060)	92	460 (164, 1041)	248	523 (92, 1207)	1.12 (0.87, 1.44)	1.13 (0.96, 1.32)
First musculoskeletal consultation to first referral, days	636	143 (43, 585)	49	223 (49, 645)	130	159 (25, 705)	1.17 (0.75, 1.79)	1.15 (0.87, 1.51)
First referral to JIA diagnosis, days	636	153 (45, 575)	49	166 (65, 419)	130	184 (49, 609)	0.74 (0.53, 1.06)	1.03 (0.82, 1.29)
Number of consultations between first musculoskeletal consultation and referral/JIA diagnosis	1191	2 (1, 4)	86	2 (1, 4)	228	2 (1, 4)	1.03 (0.87, 1.22)	0.95 (0.85, 1.06)
Inflammatory rheumatic musculoskeletal disease								
First musculoskeletal consultation to iRMD diagnosis, days	18 9102	1082 (524, 1478)	18 513	1275 (654, 1624)	40 689	1292 (658, 1646)	1.12 (1.10, 1.14)	1.13 (1.12, 1.14)
First musculoskeletal consultation to first referral, days	63 259	484 (85, 1011)	7177	401 (52, 963)	16 417	435 (56, 1023)	0.93 (0.90, 0.96)	0.99 (0.96, 1.01)
First referral to iRMD diagnosis, days	63 259	282 (81, 794)	7177	504 (147, 1091)	16 417	511 (108, 1093)	1.34 (1.31, 1.38)	1.33 (1.31, 1.36)
Number of consultations between first musculoskeletal consultation and referral/iRMD diagnosis	182 360	3 (1, 6)	17 709	3 (1, 6)	38 914	3 (1, 5)	0.96 (0.95, 0.97)	0.94 (0.93, 0.95)

Median number of days from first musculoskeletal consultation to RA/JIA/iRMD diagnosis and to first referral, and from referral to diagnosis; and the number of consultations between first (non-traumatic) musculoskeletal consultation and referral/diagnosis, and their comparison between time periods.

COVID-19: coronavirus disease 2019; IQR: interquartile range; iRMD: inflammatory rheumatic musculoskeletal disease; RR: rate ratio.

For patients with an incident diagnosis of RA, it took a median of three musculoskeletal consultations before a referral was coded (or incident diagnosis if this occurred before a referral was recorded) in each period. For patients diagnosed in the pre-COVID-19 pandemic period, the time to diagnosis from first (non-traumatic) musculoskeletal consultation was 1026 (IQR 405, 1443) days. This was less than the same measure for patients who were diagnosed in the early COVID-19 pandemic period, when the median was 1237 (IQR 470, 1596) days, rate ratio (RR) 1.11 (95% CI 1.07, 1.15. *P* < 0.001), and the late COVID-19 pandemic period, when the median was 1264 (IQR 515, 1649) days, RR 1.13 (95% CI 1.11, 1.16). There were no significant differences in time between first musculoskeletal consultation and referral between time periods. The time between referral and RA diagnosis was longest [287 (IQR 69, 932) days] for those who received a diagnosis in the late COVID-19 pandemic period, compared with 196 (IQR 63, 673) days in the pre-COVID-19 pandemic period, RR 1.27 (95% CI 1.23, 1.32, *P* < 0.001).

Supplementary Table S6a, available at *Rheumatology Advances in Practice* online, demonstrates that patterns vary according to region. The East Midlands, Yorkshire and the Humber, South West and Northern Ireland did not show significant differences between time to first musculoskeletal consultation and RA diagnosis when comparing the pre- and late COVID-19 pandemic periods. Patients residing in more deprived communities experienced longer times between first musculoskeletal consultation and RA diagnosis than those residing in the least deprived areas; and these waiting times were more greatly impacted in the late pandemic period [IMD 1: RR 1.09 (95% CI 1.04, 1.16) *vs* IMD 5: 1.16 (95% CI 1.10, 1.22)]. Similar differences were seen to a lesser extent for the time from referral to diagnosis and not observed for the time from first musculoskeletal consultation to referral.

For patients with an incident diagnosis of JIA, it took a median of two musculoskeletal consultations before a referral was coded (or incident diagnosis if this occurred before a referral was recorded), in each period. There were no significant differences between time periods for times from first musculoskeletal consultation to JIA diagnosis, from first musculoskeletal consultation to referral, or from first referral to JIA diagnosis, and this did not vary according to region or deprivation index. We are unable to present these data in tabulated form given CPRD restrictions on the minimum number of individuals that can appear in each cell (*n* = 5).

For patients with an incident diagnosis of any iRMD, it took a median of three musculoskeletal consultations before a referral was coded (or incident diagnosis if this occurred before a referral was recorded), in each period. In the pre-COVID-19 pandemic period, the median time to diagnosis from first (non-traumatic) musculoskeletal consultation was 1082 (IQR 524, 1478) days. This was less than for patients who were diagnosed in the early COVID-19 pandemic period [median 1275 (IQR 654, 1624) days, RR 1.12 (95% CI 1.10, 1.14, *P* < 0.001)] and late COVID-19 pandemic period [median 1292 (IQR 658, 1646) days, RR 1.13 (95% CI 1.12, 1.14)]. There were no significant differences between first musculoskeletal consultation and referral between time periods. The time between referral and iRMD diagnosis was longest [511 (IQR 108, 1093) days] for those who received the diagnosis in the late COVID-19 pandemic period, compared with 282 (IQR 81, 794) days in the pre-COVID-19 pandemic period [RR 1.13 (95% CI 1.31, 1.36, *P* < 0.001)].

Acknowledging variation in durations between regions, each IMD quintile from least deprived to most deprived had a longer duration for each time period (Supplementary Table S6b, available at *Rheumatology Advances in Practice* online). For example, the time between first musculoskeletal consultation and diagnosis in the late COVID-19 pandemic period was 1299 (IQR 682, 1644) days for the least deprived quintile and 1320 (734, 1666) days for the most deprived quintile.

## Discussion

Changes in health-care delivery attributable to the COVID-19 pandemic have impacted the care of patients with incident iRMDs. Proportionally, fewer referrals to musculoskeletal specialist care took place in the early COVID-19 pandemic period, then increased through the late pandemic period, although not to pre-pandemic referral rates. The time from first non-traumatic musculoskeletal consultation to referral and subsequent incident JIA diagnosis was not significantly impacted by the COVID-19 pandemic. However, the time from first musculoskeletal consultation to diagnosis and referral to diagnosis for both RA and all iRMDs was significantly impacted by pandemic practice, with residents in more deprived communities waiting longer for diagnosis.

Prevalence and incidence data for RA pre-pandemic are in line with previous reports [17] and provide additional data spanning the pandemic period, similar to those described by Russell *et al.* [18]. Reduced RA incidence in the early and late pandemic periods compared with pre-pandemic rates brings a possibility that a cohort of patients with undiagnosed RA is yet to seek health care and/or be referred for specialist care. Prevalence and incidence of iRMDs did not change materially throughout the study period, which might be explained by the heterogeneous diagnoses that composed this category, including gout and PMR, which are conditions often diagnosed and managed in primary care, with secondary care referrals in cases of diagnostic uncertainty or treatment failure. The pandemic might have altered clinician behaviour towards primary care provision of diagnosis and management instead of specialist referral. Not all patients with a diagnosis of RA/JIA had a coded referral in their primary care records. RA/JIA are typically diagnosed and managed in secondary care after fast-tracked primary care referral; this might be explained by primary care clinicians diagnosing RA/JIA themselves in the pandemic period and referring on for confirmation and definitive treatment later, or where no referral took place or it was not coded.

The pre-pandemic time from referral to diagnosis for RA was 196 (IQR 63, 673) days, which increased through to the later pandemic period [287 (IQR 69, 932) days], which might be attributable, in part, to reduced functioning departmental capacity, redeployment of staff and the cessation of biologic treatments [19]. This identified trend is important for several reasons. Firstly, there is now evidence to help emergency health-care planning in the case of future pandemics. Secondly, with the potential for an increase in referrals for new suspected RA, health-care commissioners must consider the possible future need for increased capacity to manage an increased caseload. Thirdly, a diagnosis of RA and subsequent treatment occurring further along the disease trajectory, owing to longer waiting times, has the potential for a longer-term impact upon patients’ health and wellbeing and upon the services supporting patients living with RA; further research to measure this impact would enable evidence-based decisions to be made by health-care commissioners and policy-makers regarding provision of specialist rheumatology services.

It is widely known that deprivation is linked with poorer health, including poorer healthy life expectancy [20]. This study demonstrates that residents of more deprived communities wait longer for diagnosis of RA. Our study has also found that residents of more deprived areas have been disproportionately impacted by the COVID-19 pandemic, reflecting UK mortality statistics, which demonstrate that deprivation has been associated with a higher rate of death from both COVID-19 and avoidable causes [21]. These health inequalities must be tackled on a national level, and the equalization of specialist service provision for patients with suspected RA living in deprived communities must form part of this plan.

Primary care clinicians referred patients who were subsequently diagnosed with an iRMD after an average of three musculoskeletal-related consultations over the course of a year or more. These musculoskeletal consultation codes consisted of a broad range of conditions, and it might be that patients are presenting with non-synovitic symptoms (e.g. joint pain, tendonitis, entrapment neuropathy) before the consultation that triggers the referral. Such a prodrome and patterns of increased consultation rates before an RA diagnosis have been identified in CPRD previously [22].

Our methods and results differed slightly from those of Russell *et al.* [18], who used OpenSAFELY-TPP, an alternative English primary care database, to answer similar questions around the incidence and time to referral and treatment of inflammatory arthritis during the pandemic. The times from referral to diagnosis were longer in our study, probably owing to the different definitions applied. We defined time of referral as the date a referral to a musculoskeletal specialty was coded in the patient record following the last coded consultation for a musculoskeletal condition, in contrast to Russell *et al.* [18], who defined it as ‘the last primary care assessment (virtual or in person) before the first rheumatology appointment’ for any condition. As acknowledged by the authors, it is possible that patients continued to present in primary care after their referral date, for further care. We did not, however, have access to the date patients were seen in rheumatology, nor DMARD prescribing. We were able to describe the time course and number of musculoskeletal consultations leading up to a referral. We also included all age groups, enabling JIA specifically to be studied.

The use of CPRD data affords benefits including a large, nationally representative population [15], enabling robust estimates of epidemiological trends [23]. However, as with all electronic health record research, there are several potential limitations. Diagnostic misclassification, non-attendance in primary care, variation in between general practitioner coding practices and a lack of coding altogether might all lead to an unmeasured shortfall in observed cases [23]. This study did not have linkage to other health databases, such as Hospital Episode Statistics (HES), so was reliant on the coding of communications from secondary care being recorded in the primary care record, to include the correct diagnostic code at the correct time (i.e. the date the diagnosis was made, not the date the letter was dictated or received). If this coding was not completed reliably, the accuracy of the results might be questioned. One might assume that coding practice would be uniform over the study period, but COVID-19 might have impacted on the availability and capacity of administrative staff to perform their roles over the course of the pandemic. Although the data were examined for potential health inequalities attributable to geographical region and deprivation, given the substantial amount of missing data in the electronic health-care record, we were unable to stratify by ethnicity.

Further research is required to quantify the predicted potential increase in RA diagnoses using the most recently available data to enable health-care commissioners to plan their delivery accordingly. Further research exploring the impact of the pandemic for people with iRMD according to ethnicity is required. It is possible that increased times from referral to diagnosis during the early and later pandemic periods are attributable to the mode of consultation with specialist services as the remote-by-default model was rapidly adopted. Further work to estimate associations between consultation mode and outcomes would provide evidence to support delivery of specialist services. This study has focused on RA and JIA; further work could explore the impact on pandemic practice for other conditions typically diagnosed and managed in primary and secondary care settings, including gout or PMR and SLE, respectively.

### Conclusion

The reduced incidence of RA seen during the early and late pandemic period suggests that there might be patients with undiagnosed RA who have yet to present and trigger a referral for diagnosis from primary care. This potential future pressure on specialist services might be exacerbated by the longer-term consequences of longer waits from referral to diagnosis for patients with RA during the pandemic period, particularly for residents of deprived communities, who waited longer for their diagnosis after referral than residents of more affluent areas. Health-care commissioners can use this information in future pandemic planning, in addition to during decision-making when optimizing service provision across different specialist services. Further research must be conducted to understand the impact of specialist service disruption for underserved communities, particularly around the later diagnosis of iRMDs.

## Supplementary Material

rkad044_Supplementary_Data

## Data Availability

Data may be obtained from a third party and are not publicly available. The data were obtained from the Clinical Practice Research Datalink under licence from the UK Medicines and Healthcare products Regulatory Agency. Clinical Practice Research Datalink data governance does not allow us to distribute patient data to other parties. Researchers may apply for data access at http://www.CPRD.com/. The data are provided by patients and collected by the NHS as part of their care and support. The interpretation and conclusions contained in this study are those of the authors alone.

## References

[1] van der Heijde D , Daikh DI, Betteridge N et al Common language description of the term Rheumatic and Musculoskeletal Diseases (RMDs) for use in communication with the lay public, healthcare providers, and other stakeholders endorsed by the European League Against Rheumatism (EULAR) and the American College of Rheumatology (ACR). Arthritis Rheumatol 2018;70:826–31. 29532625 10.1002/art.40448

[2] Vos T , Lim SS, Abbafati C et al Global burden of 369 diseases and injuries in 204 countries and territories, 1990–2019: a systematic analysis for the Global Burden of Disease Study 2019. Lancet 2020;396:1204–22. 33069326 10.1016/S0140-6736(20)30925-9 PMC7567026

[3] Jordan KP , Kadam UT, Hayward R et al Annual consultation prevalence of regional musculoskeletal problems in primary care: an observational study. BMC Musculoskelet Disord 2010;11:144. 20598124 10.1186/1471-2474-11-144 PMC2903510

[4] Watt T , Kelly E, Fisher R. Use of primary care during the COVID-19 pandemic: May 2021 update. 2021. https://www.health.org.uk/news-and-comment/charts-and-infographics/use-of-primary-care-during-the-covid-19-pandemic (2 October 2021, date last accessed).

[5] NHS England and NHS Improvement. Advice on how to establish a remote ‘total triage’ model in general practice using online consultations. 2020. https://www.england.nhs.uk/coronavirus/wp-content/uploads/sites/52/2020/03/C0098-total-triage-blueprint-september-2020-v3.pdf (2 October 2021, date last accessed).

[6] NHS England and NHS Improvement. Letter of preparedness. 2020. https://www.england.nhs.uk/coronavirus/wp-content/uploads/sites/52/2020/03/C0264-GP-preparedness-letter-14-April-2020.pdf (2 October 2021, date last accessed).

[7] Cabinet Office (GOV.UK). Staying at home and away from others (social distancing). 2020. https://www.gov.uk/government/publications/full-guidance-on-staying-at-home-and-away-from-others (2 October 2021, date last accessed).

[8] NHS England and NHS Improvement. Important and urgent - Next steps on NHS response to COVID-19. 2020. https://www.england.nhs.uk/coronavirus/wp-content/uploads/sites/52/2020/03/C0098-total-triage-blueprint-september-2020-v3.pdf (2 October 2021, date last accessed).

[9] NHS Confederation. Exploring referral to treatment waiting trajectories. 2021. https://www.nhsconfed.org/system/files/2021-05/Exploring-referral-to-treatment-waiting-trajectories-2021.pdf (2 October 2021, date last accessed).

[10] Dinmohamed AG , Visser O, Verhoeven RHA et al Fewer cancer diagnoses during the COVID-19 epidemic in the Netherlands. Lancet Oncol 2020;21:750–1. 32359403 10.1016/S1470-2045(20)30265-5 PMC7252180

[11] Wailoo A , Hock ES, Stevenson M et al The clinical effectiveness and cost-effectiveness of treat-to-target strategies in rheumatoid arthritis: a systematic review and cost-effectiveness analysis. Health Technol Assess 2017;21:1–258. 10.3310/hta21710 PMC5733384 29206093

[12] NICE. Rheumatoid arthritis in adults: management NICE guideline [NG100]. 2018. https://www.nice.org.uk/guidance/ng100 (2 October 2021, date last accessed).

[13] Combe B , Landewe R, Daien CI et al 2016 update of the EULAR recommendations for the management of early arthritis. Ann Rheum Dis 2017;76:948–59. 27979873 10.1136/annrheumdis-2016-210602

[14] National Early Inflammatory Arthritis Audit. https://arthritisaudit.org.uk/pages/home (9 August 2022, date last accessed).

[15] Herrett E , Gallagher AM, Bhaskaran K et al Data resource profile: Clinical Practice Research Datalink (CPRD). Int J Epidemiol 2015;44:827–36. 26050254 10.1093/ije/dyv098 PMC4521131

[16] NHS England. SNOWMED CT. https://www.england.nhs.uk/digitaltechnology/digital-primary-care/snomed-ct/ (11 November 2021, date last accessed).

[17] Scott IC , Whittle R, Bailey J et al Rheumatoid arthritis, psoriatic arthritis, and axial spondyloarthritis epidemiology in England from 2004 to 2020: an observational study using primary care electronic health record data. Lancet Reg Health – Europe 2022;23:100519. 36246147 10.1016/j.lanepe.2022.100519 PMC9557034

[18] Russell MD , Galloway JB, Andrews CD et al Incidence and management of inflammatory arthritis in England before and during the COVID-19 pandemic: a population-level cohort study using OpenSAFELY. Lancet Rheumatol 2022;4:e853–63. 36447940 10.1016/S2665-9913(22)00305-8 PMC9691150

[19] Nune A , Iyengar KP, Ahmed A et al Impact of COVID-19 on rheumatology practice in the UK—a pan-regional rheumatology survey. Clin Rheumatol 2021;40:2499–504. 33495972 10.1007/s10067-021-05601-1 PMC7832421

[20] Healthy life expectancy at birth and age 65 by upper tier local authority and area deprivation: England, 2012 to 2014. https://www.ons.gov.uk/peoplepopulationandcommunity/healthandsocialcare/healthandlifeexpectancies/bulletins/healthylifeexpectancyatbirthandage65byuppertierlocalauthorityandareadeprivation/england2012to2014 (11 April 2022, date last accessed).

[21] Office of National Statistics. Socioeconomic inequalities in avoidable mortality in England: 2020. 2022. https://www.ons.gov.uk/releases/socioeconomicinequalitiesinavoidablemortalityenglandandwales2020 (11 April 2022, date last accessed).

[22] Muller S , Hider S, Machin A et al Searching for a prodrome for rheumatoid arthritis in the primary care record: a case-control study in the clinical practice research datalink. Semin Arthritis Rheum 2019;48:815–20. 30072114 10.1016/j.semarthrit.2018.06.008

[23] Burton CL , Chen Y, Chesterton LS et al Trends in the prevalence, incidence and surgical management of carpal tunnel syndrome between 1993 and 2013: an observational analysis of UK primary care records. BMJ Open 2018;8:e020166. 10.1136/bmjopen-2017-020166 PMC6020969 29921681

