# Supplementary material for: The impact of the COVID-19 pandemic on referrals to musculoskeletal services from primary care and subsequent incidence of inflammatory rheumatic musculoskeletal disease: an observational study
Source: Rheumatol Adv Pract. 2023 May 2;7(2):rkad044. doi: 10.1093/rap/rkad044 (PMC10219787; doi:10.1093/rap/rkad044)
Supplement: rkad044_Supplementary_Data [file rkad044_supplementary_data.docx]

**Supplemental Material**

Supplementary Table S1. Referrals per 100 patients consulting with musculoskeletal conditions

| Segment Start | Segment End | MPC (95% CI) | Test Statistic (t) | P-Value |
| --- | --- | --- | --- | --- |
| April 2017 | February 2020 | 1.1 (1.0 to 1.3) | 26.9 | 0.0 |
| February 2020 | May 2020 | -16.8 (-35.5 to 7.2) | -2 | 0.06 |
| May 2020 | August 2020 | 16.8 (0.2 to 36.1) | 2 | 0.06 |
| August 2020 | October 2021 | 0.8 0.1 to 1.4) | 4.9 | 0.0 |

Supplementary Table S2. Joinpoint analysis of incidence rate per month of rheumatoid arthritis per 100,000 registered population

| Segment Start | Segment End | MPC (95% CI) | Test Statistic (t) | P-Value |
| --- | --- | --- | --- | --- |
| April 2017 | January 2020 | 0.3 (-0.1 to 0.7) | 1.6 | 0.12 |
| January 2020 | April 2020 | -13.3 (-35.4 to 16.3) | -1.0 | 0.33 |
| April 2020 | October 2021 | 1.9 (0.8 to 2.9) | 3.6 | 0.00 |

Supplementary Table S3. Joinpoint analysis of incidence rate per month of juvenile inflammatory arthritis per 1,000,000 registered population

| Segment Start | Segment End | MPC (95% CI) | Test Statistic (t) | P-Value |
| --- | --- | --- | --- | --- |
| April 2017 | January 2020 | 0.3 (-0.5 to 1.0) | 0.7 | 0.484 |
| January 2020 | April 2020 | -17.4 (-59.4 to 68.1) | -0.5 | 0.59 |
| April 2020 | October 2021 | 3.7 (1.6 to 6) | 3.5 | 0.001 |

Supplementary Table S4. Joinpoint analysis of all inflammatory rheumatic musculoskeletal disease per 100,000 population

| Segment Start | Segment End | MPC (95% CI) | Test Statistic (t) | P-Value |
| --- | --- | --- | --- | --- |
| April 2017 | October 2021 | -0.2 (-0.4 to 0.0) | -1.7 | 0.10 |

Supplementary Table S5. Crude monthly estimates for the proportion of patients with a referral following a musculoskeletal consultation in primary care and crude incidence rates of patients diagnosed with rheumatoid arthritis (RA), juvenile inflammatory arthritis (JIA), and any inflammatory rheumatic musculoskeletal disease (iRMD)

| **Month** | **Proportion of patients with a referral following a musculoskeletal consultation in primary care (%)** | **Proportion of patients with a *first time* referral following a musculoskeletal consultation in primary care (%)** | **Crude incidence of rheumatoid arthritis, per 100,000 (95% confidence interval)** | **Crude incidence of juvenile inflammatory arthritis, per 1,000,000 (95% confidence interval)** | **Crude incidence of inflammatory rheumatic musculoskeletal diseases, per 100,000 (95% confidence interval)** |
| --- | --- | --- | --- | --- | --- |
| Apr-17 | 8.9 | 2.9 | 2.5 (2.2 to 2.7) | 1.3 (0.7 to 1.9) | 19.9 (19.1 to 20.6) |
| May-17 | 9.6 | 3.2 | 3.1 (2.8 to 3.4) | 1.5 (0.8 to 2.2) | 25.5 (24.7 to 26.4) |
| Jun-17 | 9.5 | 3.2 | 3.2 (2.9 to 3.5) | 3.2 (0.6 to 1.8) | 27.5 (26.5 to 28.4) |
| Jul-17 | 9.6 | 3.2 | 3.2 (2.9 to 3.5) | 1.1 (0.6 to 1.7) | 25.4 (24.5 to 26.2) |
| Aug-17 | 9.6 | 3.2 | 2.9 (2.6 to 3.2) | 1.7 (1.0 to 2.4) | 22.8 (22.0 to 23.7) |
| Sep-17 | 9.8 | 3.3 | 3.1 (2.8 to 3.4) | 1.5 (0.8 to 2.2) | 21.7 (20.9 to 22.5) |
| Oct-17 | 10.3 | 3.6 | 3.4 (3.1 to 3.7) | 1.4 (0.7 to 2.0) | 23.1 (22.3 to 23.9) |
| Nov-17 | 10.5 | 3.5 | 3.2 (2.9 to 3.5) | 1.9 (1.1 to 2.6) | 22.5 (21.6 to 23.3) |
| Dec-17 | 10.3 | 3.4 | 2.7 (2.4 to 3.0) | 1.5 (0.8 to 2.1) | 18.4 (17.7 to 19.1) |
| Jan-18 | 10.7 | 3.6 | 3.1 (2.8 to 3.4) | 1.6 (1.0 to 2.3) | 25.3 (24.4 to 26.1) |
| Feb-18 | 11.2 | 3.9 | 2.5 (2.2 to 2.7) | 1.7 (1.0 to 2.4) | 20.1 (19.4 to 20.9) |
| Mar-18 | 11.3 | 3.9 | 3.9 (2.7 to 3.3) | 1.6 (0.9 to 2.2) | 21.9 (21.1 to 22.7) |
| Apr-18 | 11.1 | 3.8 | 3.0 (2.7 to 3.3) | 1.2 (0.6 to 1.8) | 24.7 (23.8 to 25.5) |
| May-18 | 11.3 | 4.0 | 3.3 (3.0 to 3.6) | 1.9 (1.1 to 2.6) | 25.6 (24.7 to 26.4) |
| Jun-18 | 11.4 | 4.0 | 3.4 (3.1 to 3.7) | 1.6 (0.9 to 2.3) | 26.9 (26.0 to 27.8) |
| Jul-18 | 11.6 | 4.1 | 3.8 (3.5 to 4.1) | 1.3 (0.7 to 1.9) | 29.5 (28.6 to 30.4) |
| Aug-18 | 11.5 | 4.0 | 3.0 (2.7 to 3.3) | 1.4 (0.8 to 2.0) | 24.5 (23.6 to 25.3) |
| Sep-18 | 11.7 | 4.0 | 3.0 (2.7 to 3.3) | 1.5 (0.8 to 2.1) | 21.0 (20.2 to 21.8) |
| Oct-18 | 12.2 | 4.2 | 3.5 (3.1 to 3.8) | 1.5 (0.9 to 2.2) | 24.0 (23.2 to 24.9) |
| Nov-18 | 12.4 | 4.2 | 3.4 (3.1 to 3.7) | 1.9 (1.2 to 2.7) | 22.3 (21.5 to 23.1) |
| Dec-18 | 12.0 | 3.9 | 2.8 (2.5 to 3.1) | 1.9 (1.2 to 2.7) | 19.2 (18.4 to 19.9) |
| Jan-19 | 12.5 | 4.2 | 3.2 (2.9 to 3.5) | 1.2 (0.6 to 1.7) | 23.7 (22.9 to 24.6) |
| Feb-19 | 12.6 | 4.3 | 3.0 (2.7 to 3.3) | 1.0 (0.5 to 1.6) | 22.0 (21.2 to 22.8) |
| Mar-19 | 12.7 | 4.4 | 3.4 (3.0 to 3.7) | 0.9 (0.4 to 1.4) | 23.9 (23.1 to 24.7) |
| Apr-19 | 12.6 | 4.3 | 3.3 (3.0 to 3.6) | 1.6 (0.9 to 2.2) | 23.1 (22.2 to 23.9) |
| May-19 | 12.6 | 4.4 | 3.4 (3.1 to 3.7) | 1.9 (1.2 to 2.7) | 24.6 (23.7 to 25.4) |
| Jun-19 | 13.0 | 4.4 | 3.1 (2.8 to 3.3) | 2.1 (1.4 to 2.9) | 24.8 (24.0 to 25.7) |
| Jul-19 | 13.2 | 4.5 | 3.7 (3.3 to 4.0) | 2.0 (1.3 to 2.7) | 29.3 (28.3 to 30.2) |
| Aug-19 | 12.9 | 4.4 | 3.3 (3.0 to 3.6) | 1.3 (0.7 to 1.8) | 25.4 (24.6 to 26.3) |
| Sep-19 | 12.9 | 4.3 | 3.3 (3.0 to 3.6) | 1.2 (0.6 to 1.8) | 23.7 (22.8 to 24.5) |
| Oct-19 | 13.8 | 4.6 | 3.4 (3.1 to 3.7) | 1.6 (0.9 to 2.3) | 23.8 (22.9 to 24.6) |
| Nov-19 | 13.6 | 4.4 | 3.2 (2.9 to 3.5) | 1.6 (0.9 to 2.3) | 22.1 (21.3 to 22.9) |
| Dec-19 | 13.5 | 4.2 | 2.5 (2.2 to 2.7) | 1.0 (0.5 to 1.6) | 19.7 (19.0 to 20.5) |
| Jan-20 | 14.1 | 4.6 | 3.2 (2.9 to 3.5) | 1.8 (1.1 to 2.5) | 26.7 (25.8 to 27.6) |
| Feb-20 | 14.7 | 4.8 | 3.1 (2.8 to 3.4) | 1.5 (0.8 to 2.1) | 26.1 (25.2 to 26.9) |
| Mar-20 | 14.4 | 4.3 | 2.7 (2.4 to 3.0) | 0.7 (0.3 to 1.2) | 22.6 (21.8 to 23.4) |
| Apr-20 | 9.1 | 1.4 | 1.7 (1.5 to 1.9) | 1.0 (0.4 to 1.5) | 15.6 (14.9 to 16.3) |
| May-20 | 9.5 | 2.4 | 1.6 (1.4 to 1.9) | 0.8 (0.3 to 1.3) | 17.4 (16.7 to 18.1) |
| Jun-20 | 12.2 | 3.7 | 2.4 (2.1 to 2.7) | 0.8 (0.3 to 1.2) | 22.8 (22.0 to 23.7) |
| Jul-20 | 13.7 | 4.5 | 2.4 (2.2 to 2.7) | 1.1 (0.6 to 1.7) | 23.2 (22.4 to 24.1) |
| Aug-20 | 13.6 | 4.5 | 2.0 (1.7 to 2.2) | 0.7 (0.2 to 1.1) | 22.5 (21.6 to 23.3) |
| Sep-20 | 13.7 | 4.5 | 2.6 (2.3 to 2.9) | 1.2 (0.6 to 1.7) | 20.7 (19.9 to 21.4) |
| Oct-20 | 13.9 | 4.5 | 2.6 (2.3 to 2.8) | 1.3 (0.7 to 1.9) | 20.0 (19.2 to 20.7) |
| Nov-20 | 13.8 | 4.2 | 2.8 (2.6 to 3.1) | 1.2 (0.6 to 1.8) | 20.2 (19.4 to 20.9) |
| Dec-20 | 13.9 | 4.2 | 2.3 (2.0 to 2.5) | 1.3 (0.7 to 1.9) | 18.4 (17.6 to 19.1) |
| Jan-21 | 13.1 | 3.9 | 2.5 (2.3 to 2.8) | 1.6 (0.9 to 2.3) | 17.8 (17.1 to 18.5) |
| Feb-21 | 13.3 | 4.1 | 2.6 (2.3 to 2.9) | 1.5 (0.8 to 2.1) | 19.0 (18.2 to 19.7) |
| Mar-21 | 14.2 | 4.6 | 2.9 (2.6 to 3.2) | 1.8 (1.0 to 2.5) | 23.9 (23.0 to 24.7) |
| Apr-21 | 14.0 | 4.6 | 2.6 (2.4 to 2.9) | 1.1 (0.5 to 1.6) | 21.0 (20.2 to 21.8) |
| May-21 | 14.4 | 4.7 | 3.0 (2.7 to 3.3) | 1.5 (0.9 to 2.2) | 22.2 (21.4 to 23.0) |
| Jun-21 | 14.4 | 4.7 | 2.8 (2.6 to 3.1) | 1.1 (0.5 to 1.6) | 26.1 (25.2 to 27.0) |
| Jul-21 | 15.0 | 4.8 | 3.0 (2.7 to 3.3) | 1.6 (0.9 to 2.3) | 26.1 (25.2 to 27.0) |
| Aug-21 | 15.1 | 4.9 | 2.6 (2.3 to 2.8) | 1.5 (0.8 to 2.2) | 22.5 (21.7 to 23.3) |
| Sep-21 | 15.7 | 5.0 | 3.1 (2.5 to 3.4) | 2.0 (1.3 to 2.8) | 24.1 (23.2 to 24.9) |
| Oct-21 | 15.8 | 5.1 | 2.5 (2.3 to 2.8) | 1.3 (0.7 to 1.9) | 20.4 (19.6 to 21.1) |

Supplementary Table S6a. Median number of days: from first musculoskeletal consultation to rheumatoid arthritis diagnosis and to first referral; from referral to diagnosis; and the number of consultations between first (non-traumatic) musculoskeletal consultation and referral/diagnosis, and their comparison between time periods, by region and deprivation.

| **First musculoskeletal consultation to rheumatoid arthritis diagnosis (days)** | | | | | | | | |
| --- | --- | --- | --- | --- | --- | --- | --- | --- |
| **Stratified by** | **Pre-COVID-19 (01/04/2017 – 31/03/2020) period** | | **Early-COVID-19 (01/04/2020 – 31/07/2021) period** | | **Late-COVID-19 lockdown (01/08/2020 – 31/10/2021) period** | | **Pre- vs. Early-COVID-19 period** | **Pre- vs. Late-COVID-19 period** |
|  | **n** | **Median (IQR)** | **n** | **Median (IQR)** | **n** | **Median (IQR)** | **RR**  **(95% CI)** | **RR**  **(95% CI)** |
| **Overall** | 32627 | 1026 (405, 1443) | 2510 | 1237 (470, 1596) | 5865 | 1264 (515, 1649) | 1.11 (1.07, 1.15) | 1.13 (1.11, 1.16) |
| **Region** | | | | | | | | |
| North East | 1087 | 1049 (477, 1467) | 86 | 1396 (631, 1624) | 194 | 1351 (785, 1667) | 1.22 (1.03, 1.45) | 1.17 (1.04, 1.32) |
| North West | 6060 | 1033 (416, 1442) | 536 | 1223 (412, 1632) | 1127 | 1312 (558, 1672) | 1.11 (1.03, 1.19) | 1.16 (1.10, 1.22) |
| Yorkshire and The Humber | 1089 | 1007 (414, 1449) | 73 | 1148 (220, 1656) | 167 | 1226 (500, 1673) | 1.12 (0.92, 1.36) | 1.11 (0.97, 1.28) |
| East Midlands | 520 | 983 (337, 1445) | 36 | 1260 (646, 1573) | 82 | 1335 (459, 1640) | 1.14 (0.86, 1.51) | 1.16 (0.95, 1.41) |
| West Midlands | 5410 | 1036 (437, 1449) | 364 | 1101 (407, 1524) | 964 | 1252 (541, 1669) | 1.03 (0.94, 1.12) | 1.13 (1.06, 1.20) |
| East of England | 1493 | 987 (350, 1418) | 110 | 1291 (500, 1569) | 262 | 1342 (532, 1661) | 1.11 (0.95, 1.31) | 1.18 (1.06, 1.31) |
| London | 5763 | 1026 (363, 1447) | 423 | 1135 (392, 1549) | 1034 | 1252 (520, 1636) | 1.09 (1.00, 1.18) | 1.15 (1.08, 1.21) |
| South East | 6275 | 1008 (394, 1443) | 473 | 1310 (600, 1606) | 1184 | 1206 (448, 1613) | 1.16 (1.07, 1.25) | 1.10 (1.04, 1.15) |
| South West | 4095 | 1026 (410, 1428) | 360 | 1261 (556, 1624) | 808 | 1242 (467, 1665) | 1.14 (1.04, 1.25) | 1.12 (1.05, 1.20) |
| Northern Ireland | 131 | 1113 (589, 1463) | 16 | 617 (262, 1519) | 21 | 969 (528, 1478) | 0.24 (0.16, 0.36) | 0.87 (0.62, 1.21) |
| Missing | 704 | 1045 (442, 1493) | 33 | 1526 (705, 1740) | 22 | 1211 (871, 1616) | 1.22 (0.94, 1.59) | 1.19 (0.87, 1.62) |
| **Index of Multiple Deprivation** | | | | | | | | |
| 1 (Least deprived) | 6248 | 997 (386, 1426) | 449 | 1295 (448, 1596) | 1105 | 1216 (451, 1604) | 1.15 (1.06, 1.25) | 1.09 (1.04, 1.16) |
| 2 | 6373 | 1022 (416, 1445) | 505 | 1232 (398, 1568) | 1090 | 1306 (541, 1666) | 1.08 (1.00, 1.17) | 1.14 (1.08, 1.20) |
| 3 | 6160 | 1037 (400, 1443) | 467 | 1151 (420, 1598) | 1038 | 1292 (532, 1664) | 1.07 (0.99, 1.16) | 1.14 (1.08, 1.21) |
| 4 | 6019 | 1009 (406, 1446) | 501 | 1258 (500, 1606) | 1076 | 1325 (688, 1672) | 1.12 (1.04, 1.21) | 1.16 (1.10, 1.22) |
| 5 (Most deprived) | 5768 | 1061 (440, 1465) | 444 | 1300 (673, 1623) | 1005 | 1308 (631, 1673) | 1.13 (1.05, 1.22) | 1.16 (1.10, 1.22) |
| Missing | 2059 | 1001 (359, 1419) | 144 | 1005 (279, 1548) | 551 | 982 (105, 1561) | 1.06 (0.91, 1.23) | 1.03 (0.94, 1.13) |
| **First musculoskeletal consultation to first referral (days)** | | | | | | | | |
| **Overall** | 24842 | 448 (77, 1026) | 2034 | 458 (53, 1099) | 4665 | 438 (59, 1091) | 1.02 (0.96, 1.09) | 1.03 (0.99, 1.08) |
| **Region** | | | | | | | | |
| North East | 865 | 493 (88, 1020) | 69 | 142 (44, 1080) | 156 | 471 (45, 1226) | 0.85 (0.61, 1.18) | 1.14 (0.90, 1.44) |
| North West | 4659 | 444 (75, 1020) | 440 | 496 (74, 1195) | 873 | 470 (69, 1103) | 1.09 (0.96, 1.24) | 1.05 (0.96, 1.17) |
| Yorkshire and The Humber | 834 | 470 (98, 997) | 62 | 506 (21, 1197) | 134 | 399 (44, 956) | 1.15 (0.81, 1.61) | 0.93 (0.73, 1.18) |
| East Midlands | 390 | 618 (109, 1106) | 29 | 1126 (665, 1521) | 64 | 722 (140, 1308) | 1.46 (0.92, 2.31) | 1.10 (0.79, 1.52) |
| West Midlands | 4147 | 490 (79, 1077) | 293 | 494 (61, 1138) | 779 | 469 (61, 1138) | 1.01 (0.86, 1.18) | 0.99 (0.89, 1.09) |
| East of England | 1205 | 456 (80, 993) | 92 | 343 (24, 892) | 218 | 451 (70, 1009) | 0.88 (0.67, 1.17) | 1.03 (0.85, 1.25) |
| London | 4323 | 360 (60, 491) | 329 | 349 (31, 925) | 836 | 358 (43, 1027) | 0.97 (0.83, 1.13) | 1.06 (0.96, 1.18) |
| South East | 4910 | 453 (71, 1055) | 394 | 490 (71, 1041) | 978 | 404 (56, 1061) | 1.04 (0.90, 1.19) | 1.00 (0.91, 1.10) |
| South West | 2890 | 497 (95, 1054) | 287 | 458 (71, 1141) | 593 | 512 (70, 1183) | 0.99 (0.85, 1.16) | 1.08 (0.96, 1.21) |
| Northern Ireland | 87 | 619 (176, 1116) | 8 | 146 (6, 286) | 13 | 498 (50, 878) | 0.21 (0.09, 0.53) | 0.89 (0.41, 1.90) |
| Missing | 532 | 487 (107, 1066) | 31 | 772 (203, 1361) | 21 | 399 (132, 1038) | 1.24 (0.80, 1.94) | 0.95 (0.56, 1.62) |
| **Index of Multiple Deprivation** | | | | | | | | |
| 1 (Least deprived) | 4850 | 463 (69, 1036) | 357 | 448 (63, 999) | 931 | 364 (43, 1033) | 0.99 (0.85, 1.14) | 0.95 (0.87, 1.05) |
| 2 | 4929 | 455 (85, 1040) | 418 | 485 (34, 1164) | 908 | 465 (71, 1088) | 1.04 (0.91, 1.19) | 1.03 (0.94, 1.13) |
| 3 | 4701 | 452 (76, 1030) | 373 | 411 (70, 1052) | 822 | 459 (67, 1125) | 0.99 (0.86, 1.14) | 1.05 (0.95, 1.16) |
| 4 | 4541 | 434 (77, 1012) | 410 | 459 (74, 1053) | 868 | 458 (72, 1112) | 0.99 (0.86, 1.13) | 1.06 (0.96, 1.17) |
| 5 (Most deprived) | 4380 | 448 (78, 1029) | 369 | 547 (56, 1169) | 823 | 501 (67, 1121) | 1.10 (0.95, 1.27) | 1.08 (0.98, 1.20) |
| Missing | 1441 | 376 (71, 939) | 107 | 386 (49, 805) | 313 | 332 (30, 998) | 1.01 (0.77, 1.32) | 1.01 (0.85, 1.19) |
| **Referral to rheumatoid arthritis diagnosis (days)** | | | | | | | | |
| **Overall** | 24842 | 196 (63, 673) | 2034 | 274 (64, 910) | 4665 | 287 (69, 932) | 1.23 (1.17, 1.30) | 1.27 (1.23, 1.32) |
| **Region** | | | | | | | | |
| North East | 865 | 196 (45, 753) | 69 | 771 (167, 771) | 156 | 258 (68, 994) | 1.71 (1.27, 2.32) | 1.23 (1.00, 1.51) |
| North West | 4659 | 202 (67, 685) | 440 | 219 (69, 735) | 873 | 274 (67, 1002) | 1.12 (1.00, 1.26) | 1.29 (1.18, 1.40) |
| Yorkshire and The Humber | 834 | 183 (62, 584) | 62 | 230 (25, 852) | 134 | 234 (59, 1108) | 1.12 (0.83, 1.52) | 1.35 (1.09, 1.68) |
| East Midlands | 390 | 120 (53, 407) | 29 | 141 (62, 200) | 64 | 158 (51, 776) | 0.56 (0.35, 0.87) | 1.31 (0.96, 1.80) |
| West Midlands | 4147 | 162 (58, 606) | 293 | 174 (40, 794) | 779 | 294 (73, 882) | 1.07 (0.93, 1.23) | 1.35 (1.23, 1.48) |
| East of England | 1205 | 200 (65, 604) | 92 | 408 (119, 1016) | 218 | 415 (104, 898) | 1.45 (1.14, 1.83) | 1.42 (1.21, 1.67) |
| London | 4323 | 276 (86, 822) | 329 | 456 (129, 971) | 836 | 399 (111, 1056) | 1.22 (1.08, 1.38) | 1.24 (1.14, 1.35) |
| South East | 4910 | 181 (58, 368) | 394 | 261 (50, 929) | 978 | 199 (57, 897) | 1.32 (1.17, 1.49) | 1.23 (1.13, 1.33) |
| South West | 2890 | 160 (60, 636) | 287 | 339 (53, 1011) | 593 | 224 (49, 783) | 1.38 (1.20, 1.59) | 1.19 (1.08, 1.32) |
| Northern Ireland | 87 | 239 (83, 702) | 8 | 105 (21, 199) | 13 | 234 (86, 388) | 0.29 (0.14, 0.62) | 0.85 (0.47, 1.56) |
| Missing | 532 | 172 (58, 656) | 31 | 292 (61, 673) | 21 | 672 (104, 868) | 1.18 (0.77, 1.80) | 1.54 (0.92, 2.57) |
| **IMD** | | | | | | | | |
| 1 (Least deprived) | 4850 | 161 (56, 608) | 357 | 384 (76, 980) | 931 | 253 (59, 899) | 1.43 (1.26, 1.63) | 1.31 (1.20, 1.42) |
| 2 | 4929 | 190 (61, 644) | 418 | 210 (59, 789) | 908 | 300 (63, 942) | 1.12 (1.00, 1.27) | 1.31 (1.20, 1.42) |
| 3 | 4701 | 191 (64, 661) | 373 | 183 (54, 854) | 822 | 258 (66, 909) | 1.17 (1.03, 1.32) | 1.26 (1.16, 1.37) |
| 4 | 4541 | 216 (63, 690) | 410 | 305 (100, 1010) | 868 | 313 (82, 973) | 1.31 (1.17, 1.47) | 1.30 (1.19, 1.41) |
| 5 (Most deprived) | 4380 | 212 (70, 735) | 369 | 293 (62, 888) | 823 | 322 (92, 953) | 1.17 (1.04, 1.32) | 1.26 (1.15, 1.37) |
| Missing | 1441 | 265 (82, 755) | 107 | 295 (82, 902) | 313 | 197 (57, 899) | 1.12 (0.90, 1.40) | 1.06 (0.93, 1.22) |
| **Number of consultations between first musculoskeletal consultation and referral/rheumatoid arthritis diagnosis** | | | | | | | | |
| **Overall** | 30143 | 3 (1, 6) | 2338 | 3 (1, 5) | 5486 | 3 (1, 5) | 0.92 (0.88, 0.96) | 0.92 (0.90, 0.95) |
| **Region** | | | | | | | | |
| North East | 1013 | 3 (1, 6) | 82 | 2 (1, 5) | 178 | 3 (2, 6) | 0.84 (0.68, 1.03) | 0.92 (0.79, 1.06) |
| North West | 5615 | 3 (2, 6) | 507 | 3 (2, 6) | 1082 | 3 (2, 6) | 1.03 (0.94, 1.12) | 0.91 (0.85, 0.97) |
| Yorkshire and The Humber | 1008 | 3 (2, 6) | 64 | 3 (1, 7) | 156 | 3 (1, 6) | 0.89 (0.69, 1.13) | 0.97 (0.82, 1.15) |
| East Midlands | 485 | 3 (1, 6) | 35 | 2 (1, 5) | 79 | 3 (2, 6) | 0.84 (0.60, 1.17) | 1.07 (0.85, 1.34) |
| West Midlands | 5061 | 3 (1, 6) | 337 | 2 (1, 3) | 901 | 3 (1, 5) | 0.93 (0.84, 1.03) | 0.87 (0.82, 0.94) |
| East of England | 1380 | 3 (1, 5) | 98 | 3 (1, 5) | 248 | 3 (1, 5) | 0.78 (0.64, 0.95) | 0.99 (0.88, 1.12) |
| London | 5148 | 3 (1, 5) | 383 | 2 (1, 4) | 931 | 2 (1, 4) | 0.81 (0.73, 0.90) | 0.87 (0.81, 0.93) |
| South East | 5787 | 3 (1, 5) | 442 | 3 (1, 5) | 1103 | 3 (1, 5) | 0.88 (0.81, 0.97) | 0.93 (0.88, 0.99) |
| South West | 3859 | 3 (2, 7) | 344 | 3 (1, 6) | 766 | 3 (2, 7) | 0.92 (0.82, 1.02) | 1.00 (0.92, 1.08) |
| Northern Ireland | 127 | 3 (2, 5) | 14 | 1 (1, 2) | 21 | 2 (2, 5) | 0.33 (0.14, 0.76) | 0.69 (0.43, 1.09) |
| Missing | 660 | 3 (2, 6) | 32 | 4 (2, 5) | 21 | 2 (1, 4) | 1.06 (0.79, 1.43) | 0.97 (0.68, 1.39) |
| **Index of Multiple Deprivation** | | | | | | | | |
| 1 (Least deprived) | 5811 | 3 (1, 6) | 420 | 3 (2, 6) | 1026 | 3 (2, 5) | 1.02 (0.93, 1.12) | 0.89 (0.84, 0.95) |
| 2 | 5948 | 3 (1, 6) | 461 | 3 (1, 6) | 1035 | 3 (2, 5) | 0.92 (0.84, 1.00) | 0.94 (0.88, 1.00) |
| 3 | 5688 | 3 (1, 6) | 440 | 3 (1, 6) | 979 | 3 (1, 6) | 1.00 (0.91, 1.09) | 0.92 (0.87, 0.99) |
| 4 | 5535 | 3 (1, 6) | 464 | 3 (1, 5) | 1016 | 3 (1, 6) | 0.80 (0.73, 0.88) | 0.95 (0.89, 1.01) |
| 5 (Most deprived) | 5298 | 3 (1, 6) | 422 | 3 (1, 6) | 953 | 3 (1, 5) | 0.88 (0.80, 0.97) | 0.92 (0.86, 0.98) |
| Missing | 1863 | 3 (1, 5) | 131 | 2 (1, 4) | 477 | 2 (1, 4) | 0.88 (0.74, 1.06) | 0.88 (0.79, 0.98) |

Supplementary Table S6b. Median number of days: from first musculoskeletal consultation to inflammatory rheumatic musculoskeletal disease diagnosis and to first referral; from referral to diagnosis; and the number of consultations between first (non-traumatic) musculoskeletal consultation and referral/diagnosis, and their comparison between time periods, by region and deprivation.

| **First musculoskeletal consultation to inflammatory rheumatic musculoskeletal disease diagnosis (days)** | | | | | | | | | |
| --- | --- | --- | --- | --- | --- | --- | --- | --- | --- |
| **Stratified by** | **Pre-COVID-19 (01/04/2017 – 31/03/2020) period** | | **Early-COVID-19 (01/04/2020 – 31/07/2021) period** | | **Late-COVID-19 lockdown (01/08/2020 – 31/10/2021) period** | | **Pre- vs. Early-COVID-19 period** | **Pre- vs. Late-COVID-19 period** | |
|  | **n** | **Median (IQR)** | **n** | **Median (IQR)** | **n** | **Median (IQR)** | **RR**  **(95% CI)** | **RR**  **(95% CI)** |  |
| **Overall** | 189102 | 1082 (524, 1474) | 18513 | 1275 (654, 1624) | 40689 | 1292 (658, 1646) | 1.12 (1.10, 1.14) | 1.13 (1.12, 1.14) | |
| **Region** | | | | | | | | | |
| North East | 6327 | 1085 (548, 1477) | 655 | 1335 (681, 1623) | 1342 | 1282 (693, 1652) | 1.12 (1.04, 1.20) | 1.14 (1.08, 1.20) | |
| North West | 36555 | 1096 (543, 1482) | 3810 | 1301 (680, 1632) | 8303 | 1308 (677, 1660) | 1.13 (1.09, 1.16) | 1.13 (1.11, 1.16) | |
| Yorkshire and The Humber | 7075 | 1084 (522, 1484) | 652 | 1240 (567, 1618) | 1589 | 1315 (700, 1652) | 1.08 (1.01, 1.16) | 1.13 (1.07, 1.18) | |
| East Midlands | 3404 | 1060 (513, 1461) | 302 | 1252 (732, 1604) | 733 | 1257 (532, 1630) | 1.15 (1.03, 1.28) | 1.11 (1.03, 1.19) | |
| West Midlands | 31502 | 1101 (556, 1490) | 3293 | 1277 (699, 1626) | 6700 | 1341 (738, 1656) | 1.12 (1.08, 1.15) | 1.15 (1.12, 1.18) | |
| East of England | 8665 | 1078 (530, 1479) | 807 | 1323 (646, 1654) | 1792 | 1293 (638, 1638) | 1.15 (1.08, 1.23) | 1.13 (1.07, 1.18) | |
| London | 28506 | 1050 (473, 1462) | 2612 | 1259 (557, 1620) | 6208 | 1242 (603, 1628) | 1.13 (1.08, 1.17) | 1.13 (1.10, 1.16) | |
| South East | 38393 | 1081 (522, 1476) | 3718 | 1267 (676, 1612) | 8517 | 1280 (651, 1634) | 1.12 (1.08, 1.15) | 1.12 (1.10, 1.15) | |
| South West | 23695 | 1074 (505, 1471) | 2359 | 1264 (609, 1623) | 5202 | 1281 (640, 1656) | 1.11 (1.07, 1.15) | 1.13 (1.10, 1.16) | |
| Northern Ireland | 602 | 1035 (574, 1463) | 66 | 1007 (408, 1510) | 135 | 1078 (348, 1519) | 0.95 (0.77, 1.19) | 1.00 (0.85, 1.18) | |
| Missing | 4378 | 1113 (560, 1518) | 239 | 1315 (793, 1683) | 168 | 1260 (745, 1643) | 1.15 (1.02, 1.29) | 1.13 (0.98, 1.29) | |
| **Index of Multiple Deprivation** | | | | | | | | | |
| 1 (Least deprived) | 40768 | 1076 (511, 1471) | 4146 | 1253 (644, 1619) | 8842 | 1299 (682, 1644) | 1.11 (1.08, 1.15) | 1.15 (1.12, 1.17) | |
| 2 | 38500 | 1078 (522, 1476) | 3603 | 1260 (624, 1617) | 7947 | 1290 (655, 1634) | 1.11 (1.07, 1.14) | 1.13 (1.11, 1.16) | |
| 3 | 35713 | 1085 (524, 1476) | 3470 | 1267 (667, 1619) | 7524 | 1307 (691, 1652) | 1.12 (1.08, 1.15) | 1.15 (1.12, 1.17) | |
| 4 | 32712 | 1083 (527, 1482) | 3268 | 1302 (687, 1634) | 6991 | 1321 (687, 1660) | 1.13 (1.10, 1.17) | 1.15 (1.12, 1.17) | |
| 5 (Most deprived) | 30623 | 1099 (548, 1489) | 3015 | 1336 (705, 1649) | 6493 | 1320 (734, 1666) | 1.14 (1.11, 1.18) | 1.15 (1.12, 1.18) | |
| Missing | 10786 | 1052 (490, 1463) | 1011 | 1160 (534, 1587) | 2892 | 1042 (220, 1558) | 1.09 (1.02, 1.16) | 0.97 (0.93, 1.01) | |
| **First musculoskeletal consultation to first referral (days)** | | | | | | | | | |
| **Overall** | 63259 | 484 (85, 1011) | 7177 | 401 (52, 963) | 16417 | 435 (56, 1023) | 0.93 (0.90, 0.96) | 0.99 (0.96, 1.01) | |
| **Region** | | | | | | | | | |
| North East | 2141 | 528 (104, 1001) | 207 | 430 (64, 939) | 537 | 408 (42, 1058) | 0.91 (0.75, 1.10) | 0.96 (0.84, 1.09) | |
| North West | 12189 | 474 (84, 1015) | 1522 | 402 (59, 1001) | 3243 | 504 (64, 1064) | 0.96 (0.90, 1.03) | 1.04 (0.98, 1.09) | |
| Yorkshire and The Humber | 2275 | 434 (85, 932) | 227 | 243 (13, 855) | 563 | 325 (35, 905) | 0.85 (0.70, 1.02) | 0.93 (0.82, 1.06) | |
| East Midlands | 945 | 569 (92, 1071) | 98 | 661 (184, 1007) | 217 | 454 (60, 1044) | 1.09 (0.83, 1.43) | 0.98 (0.81, 1.19) | |
| West Midlands | 9816 | 525 (96, 1037) | 1097 | 448 (89, 1004) | 2599 | 475 (73, 1107) | 0.95 (0.87, 1.03) | 1.01 (0.95, 1.07) | |
| East of England | 3062 | 544 (105, 1054) | 355 | 311 (23, 1111) | 843 | 429 (65, 945) | 0.88 (0.76, 1.02) | 0.87 (0.78, 0.96) | |
| London | 11534 | 419 (67, 950) | 1183 | 300 (35, 873) | 2985 | 343 (44, 945) | 0.89 (0.82, 0.97) | 0.97 (0.92, 1.03) | |
| South East | 12579 | 490 (83, 1023) | 1481 | 420 (67, 926) | 3473 | 428 (48, 1010) | 0.92 (0.86, 0.99) | 0.97 (0.92, 1.02) | |
| South West | 7206 | 504 (97, 1029) | 885 | 408 (52, 1004) | 1848 | 459 (66, 1081) | 0.91 (0.83, 0.99) | 1.01 (0.94, 1.08) | |
| Northern Ireland | 151 | 492 (162, 1024) | 22 | 290 (24, 468) | 40 | 518 (59, 1016) | 0.58 (0.33, 1.04) | 0.90 (0.57, 1.41) | |
| Missing | 1361 | 547 (115, 1074) | 100 | 601 (38, 1232) | 69 | 506 (86, 979) | 1.06 (0.82, 1.37) | 0.97 (0.71, 1.32) | |
| **Index of Multiple Deprivation** | | | | | | | | | |
| 1 (Least deprived) | 12961 | 493 (80, 1033) | 1619 | 358 (43, 926) | 3498 | 422 (49, 991) | 0.88 (0.82, 0.94) | 0.95 (0.90, 0.99) | |
| 2 | 12599 | 484 (89, 1011) | 1408 | 408 (50, 990) | 3134 | 432 (51, 1051) | 0.94 (0.88, 1.01) | 0.99 (0.94, 1.05) | |
| 3 | 11731 | 502 (91, 1032) | 1257 | 364 (45, 931) | 3054 | 450 (56, 1036) | 0.87 (0.80, 0.94) | 0.99 (0.94, 1.04) | |
| 4 | 11268 | 482 (84, 994) | 1234 | 430 (58, 1004) | 2942 | 443 (64, 1018) | 0.94 (0.87, 1.02) | 0.99 (0.94, 1.05) | |
| 5 (Most deprived) | 11070 | 468 (84, 994) | 1274 | 475 (93, 1008) | 2774 | 471 (69, 1063) | 1.01 (0.94, 1.10) | 1.04 (0.98, 1.10) | |
| Missing | 3630 | 447 (77, 966) | 385 | 396 (50, 909) | 1015 | 338 (37, 930) | 0.95 (0.82, 1.10) | 0.92 (0.83, 1.01) | |
| **Referral to iRMD diagnosis (days)** | | | | | | | | | |
| **Overall** | 63259 | 282 (81, 794) | 7177 | 504 (147, 1091) | 16417 | 511 (108, 1093) | 1.34 (1.31, 1.38) | 1.33 (1.31, 1.36) | |
| **Region** | | | | | | | | | |
| North East | 2141 | 304 (72, 818) | 207 | 671 (239, 1273) | 537 | 517 (103, 1115) | 1.52 (1.30, 1.79) | 1.33 (1.20, 1.48) | |
| North West | 12189 | 307 (84, 832) | 1522 | 496 (131, 1107) | 3243 | 510 (109, 1079) | 1.30 (1.23, 1.38) | 1.28 (1.23, 1.33) | |
| Yorkshire and The Humber | 2275 | 352 (100, 914) | 227 | 596 (159, 1197) | 563 | 676 (126, 1241) | 1.31 (1.13, 1.52) | 1.35 (1.22 (1.49) | |
| East Midlands | 945 | 184 (62, 548) | 98 | 286 (114, 865) | 217 | 210 (62, 939) | 1.39 (1.10, 1.77) | 1.36 (1.15, 1.62) | |
| West Midlands | 9816 | 238 (72, 728) | 1097 | 418 (132, 979) | 2599 | 469 (103, 1076) | 1.30 (1.21, 1.39) | 1.39 (1.33, 1.46) | |
| East of England | 3062 | 232 (70, 685) | 355 | 462 (137, 966) | 843 | 616 (144, 1091) | 1.41 (1.25, 1.60) | 1.55 (1.42, 1.68) | |
| London | 11534 | 328 (101, 829) | 1183 | 585 (202, 1181) | 2985 | 570 (153, 1129) | 1.37 (1.28, 1.46) | 1.33 (1.28, 1.39) | |
| South East | 12579 | 280 (78, 795) | 1481 | 568 (139, 1091) | 3473 | 517 (98, 1078) | 1.37 (1.29, 1.46) | 1.32 (1.26, 1.37) | |
| South West | 7206 | 253 (76, 783) | 885 | 435 (129, 1114) | 1848 | 456 (81, 1063) | 1.33 (1.23, 1.44) | 1.30 (1.22, 1.37) | |
| Northern Ireland | 151 | 139 (54, 623) | 22 | 181 (28, 381) | 40 | 301 (92, 507) | 0.80 (0.47, 1.37) | 1.08 (0.72, 1.64) | |
| Missing | 1361 | 269 (73, 761) | 100 | 525 (146, 904) | 69 | 487 (104, 1037) | 1.35 (1.07, 1.69) | 1.30 (0.99, 1.72) | |
| **Index of Multiple Deprivation** | | | | | | | | | |
| 1 (Least deprived) | 12961 | 254 (70, 709) | 1619 | 586 (136, 1128) | 3498 | 525 (102, 1077) | 1.45 (1.37, 1.54) | 1.39 (1.33, 1.45) | |
| 2 | 12599 | 280 (77, 801) | 1408 | 472 (134, 1012) | 3134 | 505 (98, 1100) | 1.26 (1.19, 1.35) | 1.33 (1.27, 1.39) | |
| 3 | 11731 | 264 (79, 767) | 1257 | 490 (161, 1120) | 3054 | 527 (113, 1092) | 1.40 (1.31, 1.50) | 1.36 (1.30, 1.42) | |
| 4 | 11268 | 298 (86, 811) | 1234 | 483 (129, 1072) | 2942 | 525 (129, 1117) | 1.30 (1.22, 1.39) | 1.34 (1.28, 1.40) | |
| 5 (Most deprived) | 11070 | 318 (93, 835) | 1274 | 517 (168, 1125) | 2774 | 533 (127, 1116) | 1.31 (1.23, 1.40) | 1.31 (1.26, 1.37) | |
| Missing | 3630 | 304 (91, 801) | 385 | 471 (164, 1028) | 1015 | 345 (75, 980) | 1.27 (1.13, 1.42) | 1.14 (1.05, 1.23) | |
| **Number of consultations between first musculoskeletal consultation and referral/iRMD diagnosis** | | | | | | | | | |
| **Overall** | 182360 | 3 (1, 6) | 17709 | 3 (1, 6) | 38914 | 3 (1, 5) | 0.96 (0.95, 0.97) | 0.94 (0.94, 0.94) | |
| **Region** | | | | | | | | | |
| North East | 6130 | 3 (1, 5) | 637 | 3 (1, 6) | 1271 | 3 (2, 6) | 1.03 (0.96, 1.10) | 1.04 (0.98, 1.09) | |
| North West | 35381 | 3 (2, 6) | 3646 | 3 (2, 6) | 8007 | 3 (2, 6) | 0.99 (0.97, 1.02) | 0.97 (0.95, 0.99) | |
| Yorkshire and The Humber | 6831 | 3 (2, 6) | 604 | 3 (2, 6) | 1525 | 3 (1, 6) | 1.02 (0.95, 1.10) | 1.00 (0.96, 1.06) | |
| East Midlands | 3291 | 3 (1, 6) | 293 | 3 (2, 6) | 715 | 3 (1, 6) | 1.10 (0.99, 1.22) | 0.99 (0.93, 1.07) | |
| West Midlands | 30552 | 3 (2, 6) | 3191 | 3 (1, 6) | 6412 | 3 (2, 6) | 0.92 (0.90, 0.95) | 0.97 (0.95, 0.99) | |
| East of England | 8372 | 3 (1, 5) | 761 | 3 (1, 5) | 1694 | 3 (1, 5) | 0.93 (0.87, 0.99) | 0.90 (0.86, 0.94) | |
| London | 26923 | 3 (1, 5) | 2460 | 2 (1, 4) | 5838 | 2 (1, 4) | 0.88 (0.85, 0.92) | 0.88 (0.86, 0.90) | |
| South East | 37025 | 3 (1, 5) | 3547 | 3 (1, 5) | 8121 | 3 (1, 5) | 0.95 (0.92, 0.98) | 0.93 (0.91, 0.94) | |
| South West | 23030 | 3 (2, 6) | 2278 | 3 (2, 7) | 5040 | 3 (2, 6) | 1.01 (0.97, 1.05) | 0.94 (0.91, 0.96) | |
| Northern Ireland | 590 | 3 (1, 4) | 63 | 2 (1, 4) | 128 | 2 (1, 3) | 0.91 (0.74, 1.13) | 0.72 (0.61, 0.85) | |
| Missing | 4235 | 3 (2, 6) | 229 | 3 (2, 6) | 163 | 3 (2, 5) | 0.91 (0.81, 1.02) | 0.87 (0.76, 0.99) | |
| **Index of Multiple Deprivation** | | | | | | | | | |
| 1 (Least deprived) | 39422 | 3 (1, 6) | 3946 | 3 (1, 5) | 8477 | 3 (1, 5) | 0.95 (0.93, 0.98) | 0.98 (0.96, 0.99) | |
| 2 | 37261 | 3 (1, 6) | 3458 | 3 (1, 6) | 7606 | 3 (1, 5) | 0.97 (0.94, 0.99) | 0.94 (0.92, 0.96) | |
| 3 | 34465 | 3 (2, 6) | 3332 | 3 (1, 5) | 7209 | 3 (1, 6) | 0.93 (0.90, 0.96) | 0.94 (0.92, 0.96) | |
| 4 | 31476 | 3 (1, 6) | 3139 | 3 (1, 6) | 6707 | 3 (1, 6) | 0.99 (0.96, 1.02) | 0.95 (0.93, 0.97) | |
| 5 (Most deprived) | 29423 | 3 (2, 6) | 2876 | 3 (1, 6) | 6257 | 3 (1, 6) | 0.97 (0.94, 1.00) | 0.93 (0.91, 0.96) | |
| Missing | 10313 | 3 (1, 5) | 958 | 2 (1, 5) | 2658 | 2 (1, 5) | 0.97 (0.92, 1.03) | 0.90 (0.87, 0.93) | |
